# Transient, Recurrent Central Nervous System Clinical Manifestations of X-Linked Charcot-Marie-Tooth Disease Presenting with Very Long Latency Periods between Episodes: Is Prolonged Sun Exposure a Provoking Factor?

**DOI:** 10.1155/2020/9753139

**Published:** 2020-06-27

**Authors:** Andria Tziakouri, Konstantinos Natsiopoulos, Kleopas A. Kleopa, Costas Michaelides

**Affiliations:** ^1^Department of Neurology, American Medical Center, Nicosia 2047, Cyprus; ^2^Department of Radiology, American Medical Center, Nicosia 2047, Cyprus; ^3^Neurology Clinics and Neuroscience Laboratory, The Cyprus Institute of Neurology and Genetics and Cyprus School of Molecular Medicine, Nicosia 1683, Cyprus

## Abstract

Charcot-Marie-Tooth disease is one of the most common inherited neurological disorders affecting the peripheral nervous system. The common clinical manifestations of the disease are distal muscle weakness and atrophy, often associated with a characteristic steppage gait and foot deformities. Transient acute and recurrent or chronic central nervous system manifestations, predominantly, dysarthria, dysphagia, motor weakness, and ataxia, have been recognized as a feature of the X-linked type 1 of CMT (CMTX1). The CNS symptoms occur typically in young age and often precede the clinical manifestation of the polyneuropathy. Several predisposing factors such as exercise, fever, and returning from areas of high altitude have been described as triggers of the CNS symptoms; however, in many cases, a substantial cause remains undetermined. In this report, we describe a patient with three attacks of transient CNS deficits at the ages of 11, 21, and 38 years, respectively, which were also accompanied by transient white matter abnormalities on MRI. Two of the attacks occurred after prolonged exposure to sunlight. In our knowledge, this is the first documented case with such long latency periods between CNS attacks as well as the only report describing intense sun exposure as a possible provoking factor.

## 1. Introduction

Charcot-Marie-Tooth disease (CMT) is a genetically and clinically heterogeneous group of inherited neuropathies affecting the peripheral nervous system. It is one of the most common neurological disorders with a prevalence of 1 in 2500 people, and it is characterized by progressive distal muscle weakness and atrophy with characteristic steppage gait, foot deformities (pes cavus), hyporeflexia, and sensory impairment [1, 2].

CMT is predominantly inherited in an autosomal dominant manner; however, there are 6 types of X-linked inheritance (CMTX1-6), out of which CMTX1 is the most common [3]. More than 400 mutations have been documented to cause CMTX1, all affecting the *GJB1* gene which encodes for the connexin32 protein (Cx32), a gap junction protein expressed mainly in the myelinating Schwann cells of the peripheral nerves, accounting for the main manifestations of peripheral neuropathy in CMTX1, as well as in the oligodendrocytes of the central nervous system (CNS) [2, 4, 5].

The phenotype of the disease is similar to that of other CMT1 types; however, transient CNS deficits causing stroke-like symptoms have been reported for the last 20 years in patients with CMTX1 which are also accompanied by transient white matter abnormalities on magnetic resonance imaging (MRI). The most common symptoms include dysarthria, motor weakness, ataxia, and dysphagia [1–3]. These episodes are sometimes associated with predisposing factors such as exercise, fever, and returning from areas of high altitude though in many cases a substantial cause remains undetermined [6, 7]. In many patients, these CNS manifestations even precede the diagnosis of CMTX1 [2, 8].

In this report, we describe a patient with CMTX1 presenting with three recurrent attacks of transient CNS deficits over a period of 20 years. The first episode occurred before an established CMTX diagnosis. The second and third episodes occurred after long periods of direct, strong sunshine exposure during the prolonged hours of work at a beach in Cyprus and his summer holidays on a Greek island, respectively. To the best of our knowledge, similar cases with such a long latency period between CNS attacks as well as the possibility of intense sun exposure representing a provoking factor have not been documented before in the English literature.

## 2. Case Report

A 38-year old man presented with dysarthria lasting for about one hour, two days after returning from holidays from the island of Corfu, where he had spent several days exposed to strong sunshine. The day after the presentation, the symptoms of dysarthria recurred and lasted about two hours before completely resolving. There were no other neurological symptoms such as headache, dizziness, confusion, or weakness (except from the baseline weakness due to the polyneuropathy). During his holidays, he was exposed to the sun for more hours than usual, but he did not report excessive use of alcohol or other substances.

The patient was examined on the second day of symptom appearance when he was found to be afebrile with no other signs of infection. His dysarthria had resolved, and the rest of his neurological examination was normal with the exception of the baseline weakness of the lower extremities due to his polyneuropathy.

Blood tests including inflammatory parameters as well as an immunological screen were normal and so was urine analysis and cerebrospinal fluid (CSF) analysis. CSF oligoclonal bands were absent.

**Figure 1:**
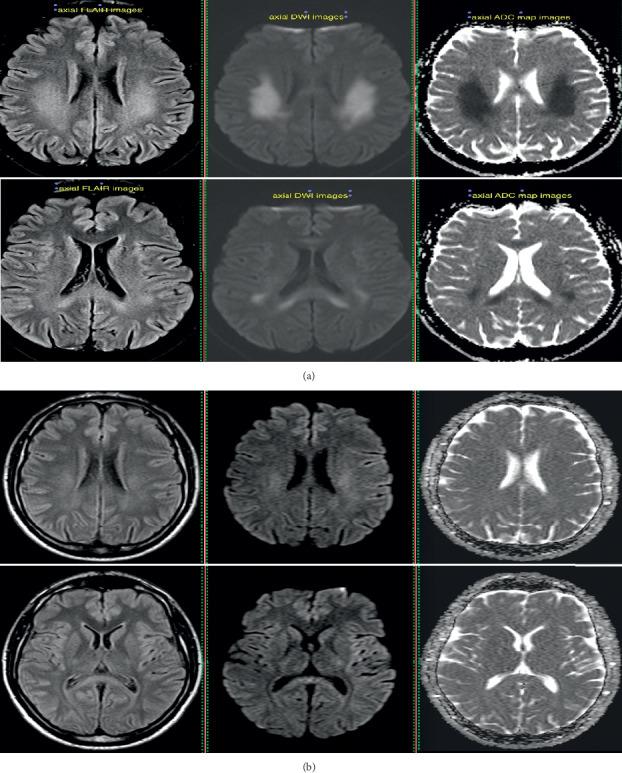
(a) Axial fluid-attenuated inversion recovery (FLAIR) and diffusion-weighted magnetic resonance imaging (DWI) upon symptom presentation showed diffuse symmetric deep white matter involvement of the centrum semiovale posterior frontal and parietal, with T2/FLAIR hyperintensity and restricted diffusion (first row of images), which extend continuously to the splenium of the corpus callosum (second row of images). (b) Follow-up MRI 1 month after presentation showed significant symmetric improvement of the findings on FLAIR and DWI images. The FLAIR abnormalities had improved both in volume and intensity; subtle increased signal could still be traced on DWI images, with no actual restricted diffusion on ADC map images.

Magnetic resonance imaging (MRI) of his brain was obtained (Figure 1) which showed a diffuse symmetric deep white matter involvement of the centrum semiovale posterior frontal and parietal, with T2/FLAIR hyperintensity and restricted diffusion an ADC map images (first row of images).

The findings extended continuously to the splenium of the corpus callosum (second row of images). No major mass effect was recognized. No hemorrhagic changes on T2/GRE images and no postcontrast enhancement on enhanced T1 images were seen (not shown here).

Due to the transient recurrence of the symptoms on the second day of presentation, the patient was treated empirically with corticosteroids (1 gram of intravenous methylprednisone per day for 3 consecutive days). His neurological examination returned to baseline.

Repeat MRI examination performed one month later showed significant symmetric improvement of the findings on T2, FLAIR, and DWI images (Figure 1). The FLAIR abnormalities were hardly recognized on the second MRI and had improved both in volume of involvement and intensity. Subtle increased signal could still be traced on DWI images, with no actual restricted diffusion on ADC map images (ADC map normalization).

Further questioning revealed two similar episodes in the past, one at the age of eleven years and one at the age of 21 years. The first episode preceded the diagnosis of CMTX, which was established at the age of sixteen, following the diagnosis of his older brother. The c.381C>G base change in the *GJB1* gene resulting in the Ile127Met amino acid substitution in Cx32 was identified in this family. He had experienced recurrent episodes of weakness of the right lower extremity lasting 2-3 hours over the course of 3 days. The medical history had revealed no evidence of infection, travel, intense exercise, or presence of any other provoking factor. MRI was not available at that time, and no medical record of laboratory examinations could be obtained.

Ten years later, at the age of twenty-one, the patient experienced a more severe episode of dysarthria lasting 4-5 hours. Over the course of the next 4 days, he continued to experience transient dysarthria attacks which subsequently progressed to paresis of the right lower limb. At that time, he had once again been exposed to strong sunshine for prolonged periods of time in the days prior to the presentation due to his work at the beach. Once again, laboratory investigation was entirely normal and he was treated with corticosteroids.

The MRI which, at that time, reported it as a possible acute disseminated encephalomyelitis (ADEM) in retrospect showed very similar findings to the one performed at our institution. The follow-up MRI was done one month later and revealed significant improvement.

## 3. Discussion

The first clinical manifestations of CMT in our patient were 2 consecutive episodes of dysarthria which occurred years before the diagnosis was established. As described in other reports, this provides evidence that CMT should be included in the differential diagnosis of transient CNS symptoms and should be investigated with an electrophysiology examination which can reveal decreased conduction velocities even before the manifestation of peripheral nervous system (PNS) symptoms [4, 6]. After a 10-year gap, our patient experienced similar episodes which progressed to severe lower right limb weakness and were associated with transient white matter abnormalities on MRI. Remarkably, the third episode occurred only 20 years later. This is to our knowledge the first patient to be documented with such a long latency period between CNS episodes. It is worth noting that neither the mother nor the brother of the patient ever experienced any CNS symptoms related to their disease. The same disease-causing mutation found in this family was previously reported by Nicholson et al.; however, no clinical CNS manifestations were described in affected patients [9].

The availability of MRI during the last 2 episodes revealed the typical association between the CNS symptoms and transient white matter abnormalities predominantly seen in the splenium of the corpus callosum extending to the centrum semiovale bilaterally [10, 11]. Restricted diffusion is also sometimes observed on MRI, and it is thought to be the result of dysfunctional gap junctions caused by Cx32 mutations. Cx32 forms gap junctions between the noncompact layers of myelin of peripheral myelinated fibers; however, growing evidence suggests that the same Cx32 mutations might disrupt the gap junction-mediated coupling between astrocytes and oligodendrocytes and along the myelin sheath formed by oligodendrocytes in the CNS, thus impairing the homeostasis of oligodendrocytes and white matter as a whole [1, 8, 12].

This is thought to occur during situations of metabolic stress. For example, proinflammatory cytokines that rise during fever or systemic illness, decreases in the pH of the CSF as seen after reacclimation from high altitudes, or hyperventilation and intense exercise are all examples of stressors thought to provoke the clinical manifestation of the CNS episodes [2, 13, 14]. The time interval seen between the stressor and the episodes can be attributed to the time needed for the gap junctions to shift their functions in order to re-establish homeostasis, thereby increasing the susceptibility to injury of Cx32-deficient oligodendrocytes in the white matter. It is suggested that, during normal circumstances, the intact gap junctions compensate for the impaired connexins [10]. However, in the absence of functional Cx32 gap junction channels, the stress-induced impairment of other gap junctions formed by astrocytic Cx43 and oligodendrocytic Cx47 result in clinical manifestations [15, 16]. Interestingly, none of the provoking factors recognized so far could be identified in our patient's case, but there was a relation between intense sun exposure for several days prior to the development of the last two episodes, something which could share the same triggering mechanism as fever in previously reported cases.

## 4. Conclusion

This case report adds to the growing literature of transient CNS manifestations seen sometimes in CMTX1 that clinicians should be aware of. It demonstrates that CNS manifestations can occur prior to the clinical manifestations of peripheral neuropathy of CMTX1 and that they can be recurrent with very long latency periods between episodes. Lastly, it highlights that prolonged exposure to strong sunshine may be a risk factor for the development of these CNS manifestations, something that had not been reported before.
